# Unapproved clinical trials in Russia: exception or norm?

**DOI:** 10.1186/s12910-021-00617-3

**Published:** 2021-04-20

**Authors:** Petr Talantov, Ravil Niyazov, Galina Viryasova, Margarita Dranitsyna, Ilya Yasny

**Affiliations:** 1 Russian Academy of Sciences Commission for Counteracting the Falsification of Scientific Research, Leninsky Prospect, 14, Moscow, Russia 119991; 2 Center for Scientific Advice Ltd., Presnensky Val, 14, Moscow, Russia 123557; 3 Belozersky Institute of Physico-Chemical Biology, Lomonosov Moscow State University, Leninskie gory, 1 build. 40, Moscow, Russia 119991; 4 Inbio Ventures, Vokzalnaya St., 11-216, Odintsovo, Russia 143007

**Keywords:** Russian clinical trials, Unauthorised clinical trials, Scientific validity of clinical trials, Unethical clinical trials, Marketing trials, Publication issues

## Abstract

**Background:**

In modern Russia, any clinical investigation of a pharmaceutical for use in humans is subject to prior evaluation and approval by the Ministry of Health and its Central Ethics Committee. Despite this, some researchers and trial sponsors fail to comply, this is particularly true in case of the studies initiated by domestic sponsors or sponsor-investigators and published in Russian language medical journals. This exploratory research aims to discover whether it is a sporadic non-compliance with regulations or a common practice.

**Methods:**

We searched the Russian language database eLIBRARY for the phrase ‘results of a randomised trial’. We selected publications reporting clinical trials and conducted in Russia. For each of the selected studies, we searched the state register of the approved clinical trials. We assessed whether (1) the investigational medicinal product was approved for marketing in Russia; (2) the therapeutic indications, posology, and administration method in the clinical trial were consistent with the approved labelling; (3) the issue of the journal included an advertisement of the medicinal product in question; and (4) the full description of the methodology corroborated that the clinical trial was randomised, as was stated in the title or abstract.

**Results:**

Of the 26 selected articles, 22 reported the results of unauthorised clinical trials. Three of those trials were conducted in children. Twenty-one studies reported on data from unauthorised trials for investigational products approved for marketing in Russia. However, in nine cases, the therapeutic indications, posology, or administration method did not match the conditions indicated in the labelling. Moreover, in one case, the unauthorised trial included a drug therapy intervention where the active substance was not approved for use in any medicinal product marketed in Russia. In 14 of the 26 articles, the issue of the journal or the article itself contained an advertisement for the same medicinal product or, in one case, its manufacturer. All publications accompanied by advertisements claimed that the medicinal product in question was efficacious.

**Conclusions:**

A substantial fraction of the clinical trials initiated by domestic sponsors and reported in Russian medical journals failed to obtain the mandatory prior evaluation and approval from the regulator. This can affect the rights and well-being of the study participants and the scientific validity of the studies.

## Background

When performing clinical studies in humans, there is an inherent risk that the interests of researchers or sponsors of the trial will prevail over the interests of the study subjects and society. There have been previous clinical trials, of which some resulted in tragic consequences, that led to death or unreasonable suffering of the participants. For a long time, scientific experiments involving humans were not regulated; therefore, the safety and rights of trial participants were largely out of consideration, and there was no responsibility for neglect. Only after dramatic episodes such as the experiments of Nazi doctors on concentration camps prisoners, the study of the natural course of syphilis in Tuskegee, or attempts to implant cancer tumours into patients at Memorial Sloan Kettering Cancer Center [1–4], drew public attention, did the situation gradually begin to change. The principles regulating this field of research emerged in the middle of the twentieth century; the research ethics guidelines were published, and changes to the legislation were made in most countries of the world. Ethical principles were first articulated in the Nuremberg Code and then elaborated in the Declaration of Helsinki [5] and are reflected in the Good Clinical Practice regulations (GCP), the latter being the international standard for clinical trials in human subjects [6].

In modern Russia, clinical investigations of pharmaceuticals in human subjects are regulated by Federal Law of 12 April 2010 FZ-61’On circulation of medicinal products’ [7] as amended and Order No 200n of 1 April 2016 of the Ministry of Health (MoH) of the Russian Federation ‘On Approval of Rules for Good Clinical Practice’ [8]. Federal law FZ-61 establishes an authorisation procedure for a clinical trial of a medicinal product for human use. The authorisation is granted by the MoH subsequent to the favourable scientific and ethical evaluation. The procedure of granting authorisation for a clinical trial on human subjects is aimed at the following:

- Respecting the rights of participants;
- Protection of their health, including by providing health insurance to the participants; and
- Verification of the scientific validity of the study based on non-clinical tests and previous clinical investigations.

The legislation defines a clinical trial as ‘any investigation of medicinal products for diagnostic, therapeutic, prophylactic, pharmacologic properties in a human subject, an animal, including their absorption, distribution, transformation and elimination, using scientific methods, to provide evidence of the safety, quality and efficacy of the medicinal product, data on the adverse reactions in humans, animals when the medicinal product is used and on the effects on drug-drug or drug-food interactions or drug-feedstuff interactions’ [7]. It is important to emphasise that any study of a pharmaceutical intended to investigate any of its properties in human subjects, whether pre- or post-approval (including phase 4 studies), is considered a clinical trial and is thus subject to authorisation by the MoH. Moreover, the definition applies for clinical trials in both humans and animals (veterinary medicines), adding an additional layer of ambiguity.

The approval procedure is performed in the following two steps: scientific aspects of the trial are reviewed by an organisation governed by the MoH, and ethical aspects are scrutinised by the single Ethics Committee. Upon authorisation, the Register of the approved clinical trials operated by the MoH is updated to indicate a new approval within several days. It is important to distinguish the opinion of the Ethics Committee that is indispensable for the clinical trial authorisation from that of local ethics committees (LECs) that operate at the level of a clinical trial site. In Russia, the legislation does not confer a regulatory power on LECs; moreover, LECs are completely excluded in the primary law [7]. Although LECs exist and review clinical trial applications, they do not play an essential role in the clinical trial approval process according to the Russian regulations. Thus, only the Ethics Council of the MoH may provide a legally binding opinion on the ethical aspects of a clinical study, and such an opinion is provided only within the clinical trial authorisation procedure in parallel with the scientific review. Then, the MoH produces a single decision based on both the outcome of the scientific review and the opinion of the Ethics Council. LEC opinion is not taken into account.

Based on the above, the existence of the favourable opinion given by an LEC does not mean that, and should not be understood as, a clinical study is approved. It should also be noted that due to the lack of transparency of LECs’ operations and the absence of regulatory oversights of their performance, it is not possible to determine whether the ethical acceptability and scientific value of the testing on human subjects are rigorously evaluated. From the GCP perspective, while the ethical review of a clinical trial needs to be independent, an LEC established within the research institution, which is not subject to a rigorous oversight by a regulatory authority, may not satisfy this GCP requirement, as it is difficult to ensure that no conflict of interests exists.

The aspiration for the present study arose after the publication of study results in a Russian medical journal for an investigational gene therapy product that was neither approved for marketing nor authorised for studying in humans, by researchers in the Russian city of Kazan [9]. We did not find any entry for this clinical trial in the Register of clinical trials approved in the Russian Federation; therefore, it is reasonable to conclude that the MoH has not evaluated it and its commencement has not been authorised. We also could not identify any publications on prior non-clinical tests of this agent, nor did we receive any clarifications or explanations after a request was sent to the authors. In this regard, we considered it crucial to assess whether it was a sporadic non-compliance with the regulations or a common practice in Russia to conduct domestic clinical investigations without prior approval.

## Methods

**Fig. 1:**
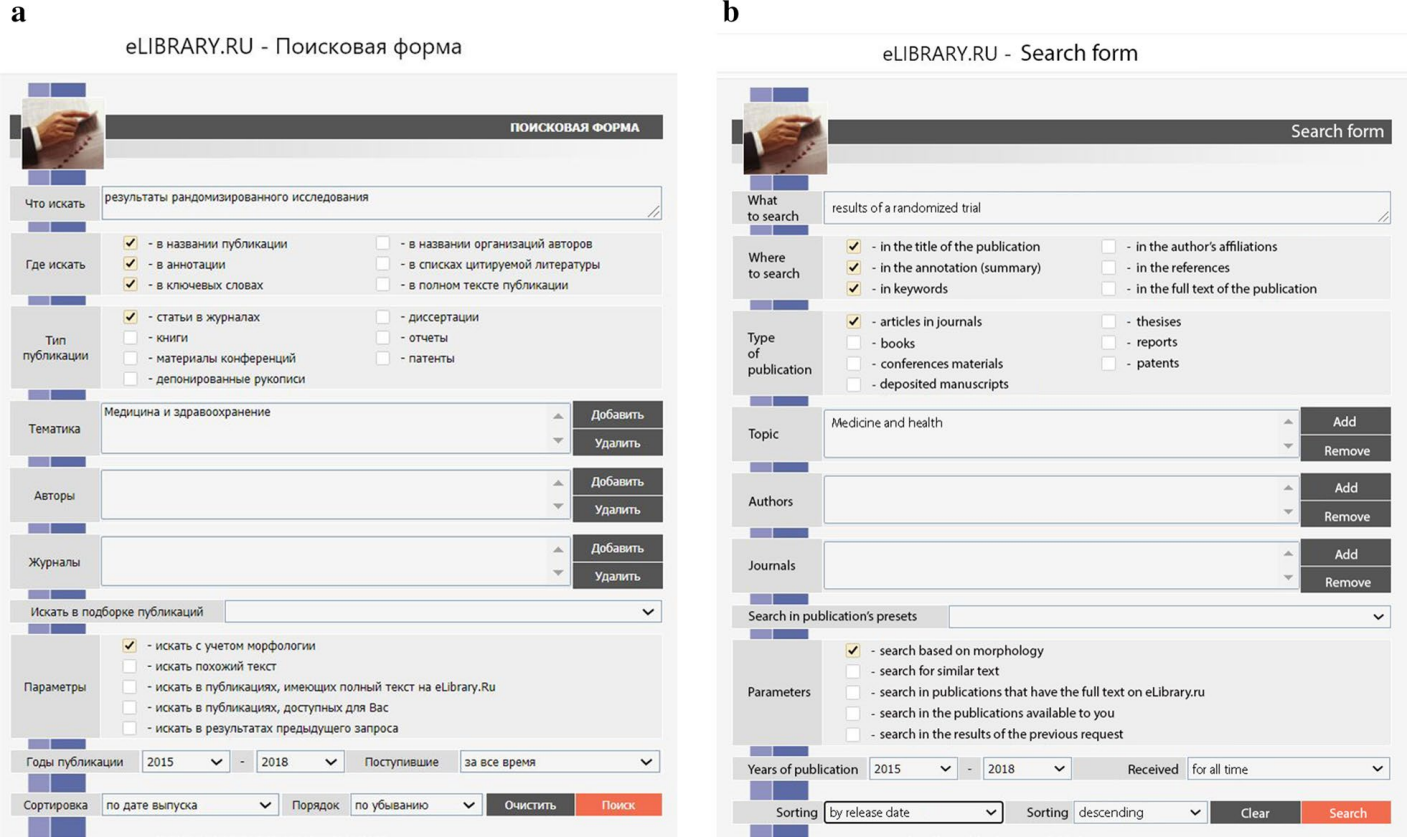
Search criteria in eLIBRARY.RU database at the inception of this study. **a** Original search form in the Russian language. **b** Search form in the English language (translated by the authors)

To assess the compliance of clinical researchers in Russia with the mandatory requirement for obtaining a clinical trial authorisation from the MoH, we searched the electronic library eLIBRARY (www.elibrary.ru), an online database of scientific articles and publications in the Russian language. While any research involving the use of pharmaceuticals in humans requires prior authorisation from the MoH, we chose to focus on clinical trials (or interventional clinical studies), i.e. studies in which the choice of a particular therapeutic strategy was preceded by assignment of the patient to a certain study group. The search phrase used was the Russian equivalent of ‘results of a randomised trial’. Because it is common in Russian medical articles to confuse observational studies with clinical trials, the only effective manner to filter out numerous observational studies was to focus on publications that stated that the study was randomised. Due to the exploratory nature of the research, we did not aim to collect a large sample or include all clinical trials within a given period of time. Our primary goal was to determine whether the problem exists, rather than to demonstrate the prevalence of violations in quantitative terms. Therefore, we assumed that it was more important to minimise false positives rather than omit false negatives. The search was performed within the ‘Medicine and Health’ section of eLIBRARY. We searched the title, abstract, and keywords of journal articles published from 2015 to 2018 with the Morphology checkbox checked (Fig. 1).

**Fig. 2:**
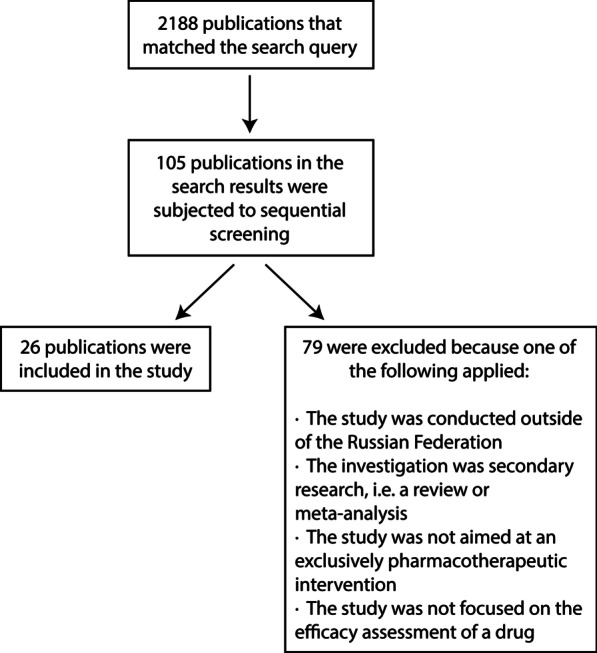
Study flow chart

We sorted the publications by date from new to old, reviewed the publications, and selected those that described human clinical trials conducted in Russia. We limited the analysis to studies on medicinal products. Surgical, anaesthetic, physical therapy, and combined methods, as well as any secondary research, including reviews and meta-analyses, were excluded. We sequentially reviewed each publication until 20 articles that met our inclusion criteria were accrued [10–29]. When we replicated the search a few months later, the results in eLIBRARY were different, in that six additional articles that met the inclusion criteria were found; these were also added to our analysis [30–35]. Having consulted the eLIBRARY staff, we could not reliably determine the cause of discrepancy in the search results at different times. The search and selection flow chart is presented in Fig. 2. Data used in the study are publicly available.

For each of the selected studies, we searched by the name of the medicinal product and, where applicable, by the active substance using the fields ‘Name of the medicinal product’ and ‘Protocol title’ in the state Register of approved clinical trials of the Russian Federation [36]. In cases where we could not find the entry in the Register, we contacted the authors and asked them to provide the details of the authorisation granted by the MoH.

In addition, we assessed the following:

- Whether the medicinal product was approved for marketing in Russia;
- In case of approved products, whether the therapeutic indications, posology, and administration method in the clinical trial under consideration were consistent with the approved labelling;
- Whether the issue of the journal in which the article was published included an advertisement of the medicinal product in question, and
- Whether the description of the methodology in the full publication corroborated that the clinical trial was randomised, as was stated in the title or abstract.

It was not appropriate or possible to involve patients or the public in the design, conduct, reporting, or dissemination plans of our research.

## Results

The articles selected for the final analysis are presented in Table 1. 22 articles [10–14, 16–19, 21–24, 26–31, 33–35] of the 26 reported the results of unauthorised clinical trials. Moreover, one of the 22 unauthorised trials was conducted in children aged from 6 to 29 months [10], and two were conducted in children aged 7–18 years [14, 33]. This is a serious issue because children are a vulnerable population, and their rights and safety need additional protection. In one case of the 26 studies, we were unable to unambiguously link the publication with the entry in the Register, and the author of the article did not respond to our request [20]. In the remaining three cases, there was a valid authorisation granted by the MoH.

**Table 1:** The analysis of the 26 publications matching the search criteria

No	Journal article	Approved indications	Approved conditions of use	Clinical trial approved by the MoH	Randomisation	Advertising	Duplication
1	Comparison of clinical efficacy and safety of the two drugs in the therapy of symptoms of eruption of infant teeth (“Dantinorm Baby®” vs “Calgel®””) [10]	+	+	−	+	+	−
2	Nonsteroid anti-inflammatory drugs for the treatement of osteoarthrosis: comparative efficacy and tolerability of nimesulide [11]	+	+	−	+	+	−
3	Efficiency of butyric acid and inulin in patients with irritable bowels syndrome: results of multicenter study [12]	−	−	−	+	+	−
4	Efficiency of different schemes of antihelicbacter therapy in patients with chronic gastroduodenal diseases and type 2 diabetes mellitus [13]	−	−	−	−	−	No. 12
5	Enterosorption in the treatment of duodenal ulcer in children in the settings of Helicobacter pylori infection and Candida colonization [14]	−	−	−	+	−	No. 24
6	Antihypertensive efficacy of chronopharmacotherapeutical approach to arterial hypertension in post transient ischemic attack patients [15]	+	+	−	+	+	−
7	Evaluation of the Reactogenicity, Safety and Immunogenicity of the Domestic Influenza Inactivated Split FLU-M Vaccine in Immunization Adults aged 18-60 Years [16]	+	+	+	+	−	−
8	Vitabact in the treatment of inflammatory diseases of eyes in the conditions of neuro reanimation [17]	+	−	−	−	−	−
9	Open prospective randomized study of the results of using Venarus in postthrombotic disease [18]	+	+	−	+	+	−
10	Clinical evaluation of the efficacy of azoxime boviolaluronidase in the prevention of excessive scarring after surgical treatment of glaucoma [19]	+	−	−	+	−	−
11	Study of clinical efficacy of original and generic drugs of ivabradine in patients with stable angina (comparative study)[20]	+	+	?	+	+	−
12	New aspects of eradicative anti-helicobacter pylori in type 2 diabetic patients with chronic gastroduodenal disorders [21]	−	−	−	−	−	No. 4
13	Estimation of hypolipidemic psillium effect in gastroenterologic patients with lipid metabolism disorders [22]	−	−	−	−	−	−
14	The inhalation therapy of moderate forms of acute bacterial rhinosinusitis [23]	−	−	−	+	+	−
15	Experience of the usage of Tonsilgon® N in the complex treatment of chronic tonsillitis with irrigations of palatine tonsils in adults [24]	+	+	−	+	+	−
16	Efficiency and safety of chemotherapy regimen with SQ109 in those suffering [25]	−	−	+	+	+	−
17	Anhedonia, depression, anxiety, and craving in opioid dependent patients stabilized on oral naltrexone or naltrexone implant [26]	+	+	−	+	−	−
18	Alcohol withdrawal syndrome dynamics during treatment with Nooclerin (deanoli aceglumas) [27]	+	+	−	+	+	−
19	Indicators’ dynamics of electrophysiological non-homogeneity of myocardium on the patients’ intensive statinology in postinfarctic period [28]	+	−	−	−/+	−	−
20	Results of the intermittent regimen of initial pain therapy with chondroitin sulfate and glucosamine sulfate for patients with osteoarthritis, back pain and comorbidity [29]	+	+	−	+	+	−
21	Observational study of the efficiency and safety of the use of tenoxicam in dorsalgy in comparison with meloxicam and [30]	+	+	−	+	+	−
22	Evaluation of efficacy and safety of rebamipide use in the triple therapy for *Helicobacter pylori* eradication: a pilot study [31]	+	+	−	+	+	−
23	Immunogenicity of the third generation hepatitis b vaccine (pre-S1/pre-S2/S) [32]	−	−	+	+	−	−
24	Enterosorption in the treatment of duodenal ulcer in children in the settings of helicobacter pylori infection and candida colonization [33]	−	−	−	+	+	No. 5
25	Effectiveness of the tenoxicam in patients with ankylosing spondylitis [34]	+	+	−	+	−	−
26	Efficiency of combined antihypertensive therapy in patients with arterial hypertension and obesity depending on the polymorphism of CYP2C9 gene [35]	+	+	−	−/+	−	−

We used the original English titles and did not correct the spelling. Column "Randomisation" can contain "+" for a clinical trial that was randomised, “−" for a clinical trial claimed to be randomised while in fact it was not, "+/−" if the randomisation was not related to the objectives of the study and the results obtained. In all studies except No. 3, 16, 23 the medicinal product of interest was approved for marketing in Russia. “Duplication” column shows if the same results at least partially were reported in another article

Twenty-one of the 22 publications reported data from unauthorised clinical trials using investigational products approved for marketing in the Russian Federation. However, in nine cases [13, 14, 17, 19, 21–23, 28, 33] of these twenty-one, the therapeutic indications, posology, or administration method did not match the conditions indicated in the labelling that was in force at the time when the clinical trial was conducted. In one case [12] of the 22 studies, the unauthorised clinical trial included a drug therapy intervention where the active substance was not approved for use in any medicinal product marketed in the Russian Federation, which indicates that the MoH did not review the scientific plausibility and safety of any clinical use of this active substance in human subjects.

In 14 [10–12, 15, 18, 20, 23–25, 27, 29–31, 33] of the 26 cases, the issue of the journal in which the article was published, or the article itself, contained an advertisement for the same medicinal product or, in one case, its manufacturer. All publications accompanied by advertisements claimed that the medicinal product in question was efficacious for the condition under study.

Although all reviewed studies were reported as randomised, in four cases [13, 17, 21, 22] of the 26 studies, no randomisation was in fact conducted, which could be recognised from either the description of the clinical trial methodology or by comparing different articles reporting the same trial. For example, one study presented as randomised compared three groups of patients who were allocated to one of the groups based on their diagnosis. Thus, three incomparable groups, with a different diagnosis in each, were created [17]. In yet another trial, different treatments were allocated to patients according to the estimated risk of cardiovascular disease [22]. In two cases [28, 35] of the 26 studies, the described randomisation process was not in line with the objectives of the study and further analysis because the study conclusions were based on comparisons of other treatment arms that were not envisaged by the randomisation scheme.

## Discussion

All clinical trials reviewed in this study were subject to prior authorisation by the MoH of the Russian Federation before commencing. We found that in at least 22 of the 26 reviewed publications, the reported trials failed to comply with the mandatory rules for clinical trial authorisation, which could have resulted in inadequate ethical and scientific review to ensure the safety of the study participants and scientific integrity of the study. Although obtaining an authorisation from the regulatory authority does not protect from malpractices, the lack of such preliminary clearance considerably increases the risk of both scientific and ethical violations.

Moreover, some of the reviewed articles contained noticeable misstatements. Specifically, in four cases, the authors claimed that their study was randomised, whereas after careful examination of the full texts described in the last paragraph of the Results section, we established that randomisation had not been performed. We suspect that either the authors of those studies might have misunderstood this term or this could be a case of intentional manipulation to make the research appear more credible for the reader. Both situations negatively affect the credibility of the studies. It is also possible that more inconsistencies would have been found if we had the opportunity to compare the text of the publication and the real data obtained in the trial. These findings also cast doubt on the quality of peer review. This warrants further research into both the quality of domestic clinical trials in Russia and the standards of reporting and review in Russian medical journals.

We assume that in some cases, the clinical studies were so-called ‘marketing trials’ [34, 35, 37], i.e. they were conducted not to answer a scientific question but to boost sales by publishing articles in professional journals. The presence of commercial advertising of the same drug product in the same issue of journals in which the articles were published reinforces our hypothesis. In such trials, the risk of conscious or unconscious distortion of the design, analysis, or interpretation of study data to make the product under study look credible is especially high [38, 39]. Therefore, the International Committee of Medical Journal Editors recommendations clearly state that ‘best practice prohibits selling advertisements intended to be juxtaposed with editorial content on the same product’ [40]. In one of the publications the authors concluded that the investigational product was efficacious, despite there being no statistically significant difference between the compared treatment arms [31]. The design of our study does not allow for the evaluation of the prevalence of marketing trials in Russian peer-reviewed biomedical journals. We want to draw the attention of prospective researchers to this issue requiring further investigation.

In our small sample, we found several instances of multiple publications. In one case, two articles described the same study and had almost completely overlapping content [14, 33]. In yet another case, two articles described the same study, wherein the first one described four treatment arms [13] and the second one mentioned only three of them [21]. We did not specifically focus on the practice of multiple publications and did not compare selected publications with other articles by the same authors. Considering the accidental findings reported in this work and the existing practice of financial incentives for publications in some universities, the scale of the malpractice can be significant and may require additional exploration.

## Limitations

Our findings warrant further research. The small sample size and conservative methodology used in our study do not allow drawing quantitative conclusions. While it is unlikely that the obtained sample is considerably skewed, a larger sample may return a different ratio of approved to unapproved clinical trials. Future researchers may choose to utilise a different search strategy by combining the results of a wider range of search queries. For instance, searching for ‘results of controlled trials’ or ‘results of comparative studies’ may fetch those clinical trials that were not randomised. To distinguish those trials from observational studies, future researchers should obtain and carefully read full texts of each article and, whenever this is insufficient, contact the authors for clarifications.

It should be noted that it is more important to establish the cause of the problem, to describe the typical researcher and the sponsor of such a trial. For example, a pivotal trial to be included in the marketing authorisation application for the pharmaceutical in question is more likely to be applied for an approval by the MoH, while a marketing trial is less. Future investigators may consider interviewing sponsors and other stakeholders involved in unapproved clinical trials to determine the underlying causes of this non-compliance issue. Our limited communication with the authors suggests that they could be unaware of the requirement to obtain prior authorisation from the MoH or they relied on contract research organisations to do the paperwork. Confusing regulations and lack of enforcement from the MoH may also have played a role.

## Conclusions

A substantial fraction of clinical trials, conducted in Russia and published in Russian language medical journals, violates the legally binding authorisation procedure and does not undergo an independent scientific or ethical review prior to trial commencement. This can affect the protection of the rights and well-being of the study participants and scientific validity of the conclusions drawn from such investigations. Publications of clinical study results in Russian journals frequently do not meet common standards and contain large evidence of ethical, scientific, and publishing breaches.

## Data Availability

All data generated or analysed during this study are included in this published article.

## Abbreviations

GCP: Good clinical practice
LEC: Local ethics committee
FZ: Federal’nyi Zakon (Federal Law)
MoH: Ministry of Health
